# Long-Term Clinical Outcomes for Adolescent and Young-Adult Uveal Melanoma Patients Treated with Dedicated Particle-Beam Radiation †

**DOI:** 10.3390/cancers17122042

**Published:** 2025-06-19

**Authors:** Carly Zako, Arina Nisanova, Vivian Weinberg, Jessica Scholey, Carisa Swason, Armin R. Afshar, Jeanne Quivey, Inder K. Daftari, Tony Tsai, Susanna S. Park, Michael Seider, Robert N. Johnson, Devron H. Char, Kavita K. Mishra

**Affiliations:** 1 Department of Radiation Oncology, University of California San Francisco, San Francisco, CA 94115, USA; 2 Department of Ophthalmology & Vision Science, University of California Davis, Sacramento, CA 95817, USA; 3 Tumori Foundation, San Francisco, CA 94114, USA; 4 Ocular Oncology Service, Department of Ophthalmology, University of California San Francisco, San Francisco, CA 94115, USA; 5 Retinal Consultants Medical Group, Sacramento, CA 95825, USA; 6 Department of Ophthalmology Kaiser Permanente Medical Center, Los Angeles, CA 90027, USA; 7 West Coast Retina Medical Group, San Francisco, CA 94109, USA

**Keywords:** uveal melanoma, UM, proton-beam radiation therapy, PBRT, helium-ion radiation, local control, LC, outcomes, adolescent and young adult, AYA

## Abstract

**Background:** Limited long-term data exist regarding post-particle-beam radiation (RT) for adolescents and young adults (AYA) with uveal melanoma (UM), and hence an in-depth analysis of clinical outcomes is presented. **Methods:** We identified AYA patients with UM (aged 13 to 45) treated with proton-beam radiation (PBRT, dose 56 GyE, *n* = 240) and helium-ion RT (median dose 70 GyE, *n* = 78). Patients were analyzed for local control (LC), eye preservation, distant metastasis (DM), and overall survival (OS) with univariate and multivariate analysis. **Results:** For the PBRT cohort, the median age and follow-up were 38.3 years (13.3–45.9) and 85.7 months (3–301), respectively. The 15-year (y) LC rate was 94%. Ciliary body (CB) tumors showed lower LC (*p* = 0.01). Largest tumor diameter (LTD) was the only independent predictor of LC (hazard ratio (HR) = 1.19, *p* = 0.017)). The 10-year eye preservation was 83%. Improved eye preservation was noted with a ≤50% dose delivered to the lens (*p* = 0.004), disc (*p* = 0.001), and nerve (*p* = 0.0004), and <20% of CB volume (*p* = 0.0002). Decreased tumor height (HR = 1.12, 95% CI: 1.01–1.2, *p* = 0.036), dose < 20% to CB (HR = 1.04, 95% CI: 1.01–1.06, *p* = 0.0002), and tumor–disc distance > 2 mm (HR = 0.27, *p* = 0.0003) were independent predictors of eye preservation. The 15-year DM-free rate was 73%, and the 15-year OS rate was 76%. The T category was the most significant predictor of DM (*p* < 0.0001), followed by LTD (*p* = 0.04). For the Helium cohort, median age and follow-up were 37.1 years (18.1–45.5) and 256.7 months (24–450), respectively. The Helium cohort 15-year LC was 99%, with 82% 10-year eye preservation. The 15-year DM-free and OS rates for Helium patients were both 83%. **Conclusions:** Excellent long-term LC and eye preservation can be seen in AYA UM patients treated with proton and helium-ion radiation. AYAs with UM appear to have better DM-free and overall survival rates than older patients with UM. Specific clinical and dose–volume parameters can help improve treatment planning and prognosis for younger patients with UM.

## Simple Summary

Uveal melanoma (UM) is a rare eye cancer, especially in adolescent and young-adult patients (AYAs), and it can significantly impact vision, quality of life, and survival. This study examines the long-term clinical outcomes of AYA UM patients (aged 13 to 45) treated with proton-beam (PBRT) and helium-ion radiation. We aim to determine the effectiveness of these treatments by measuring clinical outcomes, including local control, eye preservation, distant metastasis (DM), and overall survival. The results confirm excellent long-term local control and eye preservation (15 years: 94% PBRT and 99% Helium cohort) and demonstrate better DM-free and overall survival rates at 15 years in AYAs than older UM patients.

## 1. Introduction

Uveal melanoma (UM) is a rare tumor and a challenging diagnosis for adolescent and young-adult (AYA) patients, as it can threaten vision, quality of life, and life expectancy. Radiation therapy (RT) with charged particles, including proton (PBRT), helium, and carbon ion therapy, as well as plaque brachytherapy and stereotactic photon techniques, is the standard of care for UM [1]. Particle-beam radiotherapy with dedicated ocular beamlines is associated with excellent local tumor control and eye preservation due to the uniform dose distribution, minimal scatter, and sharp dose fall-off near critical structures [1]. Survival rates post-radiotherapy are similar to those observed with enucleation [2]. However, limited long-term data exist post-particle-beam radiation specifically for AYA UM patients, with few studies detailing clinical characteristics in younger individuals [3,4,5,6,7,8].

UM is the most common primary intraocular tumor in adults, occurring in older Caucasian individuals between 60 and 79 years of age [5,9,10]. The annual incidence is estimated at 5–9 new cases per million persons in the US and Europe [11]. Uveal melanoma is rare in younger populations, with children and teenagers representing approximately 1% of all cases [12,13]. The annual incidence of UM increases with age per million population: from 0.2 for ages 10–14 to 1.0 for ages 20–24, 3.4 for ages 30–34, and 6.3 for ages 40–44 [14]. The rates jump significantly thereafter to 17 for ages 50–54, 26.6 for ages 60–64, and 42.3 for ages 70–74 [14]. Across the cancer continuum, there is an increasing awareness of the differences in medical, psychological, and social needs and challenges for younger-aged cancer patients and the gaps in knowledge regarding AYA oncology [15,16]. The AYA age cutoffs vary from 24 to 50 years for “young adults” in different cancer studies [15,17,18]. For these reasons, the AYA cutoff in our cohort was set at 45 years old.

Previous UM reports studying children and teenagers found similar outcomes to older cohorts, though metastatic prognosis is improved for very young patients (<16–20 years) [4,6,10,12,19,20]. A study of 8033 eyes with UM found smaller tumor dimensions and lower tumor-related metastasis rates at 10 years in patients under 20 years old (9%) than those aged 21–60 (23%) and over 60 years old (28%) [10]. An examination of 43 patients aged <21 revealed superior metastatic control and survival rates compared to matched adult counterparts (*n* = 129), with comparable responses in eye retention and preservation of functional vision observed in both age groups [3]. One recent study with proton therapy for patients 15–39 years old and matched adults over 40 years of age reported that local tumor control, metastasis incidence, and overall survival were similar in both groups [11].

The aim of this analysis was to report long-term clinical outcomes and patient-tumor-planning characteristics for adolescent and young-adult UM patients aged 13 to 45 years undergoing particle-beam treatment, to better inform patients and clinical teams. To our knowledge, this report is among the few long-term studies of post-proton treatment and is the first study of helium ion to focus on this younger UM age group.

## 2. Materials and Methods

### 2.1. Patient Population

Patients were identified in a single institution’s prospectively maintained database (*n* = 2558) of eye treatment with proton-beam radiation (PBRT, 1994–2020) and helium-ion RT (He^+^, 1979–1992). All included patients were treated for non-metastatic UM. The exclusion criteria included age below 13 or above 45 at the time of RT, subtherapeutic RT dose, and patients treated with post-local eyewall resection or salvage RT. AYA patients were aged 13 to 45 years (i.e., not yet turned 46 years of age) at the time of radiation, resulting in 78 helium-ion RT and 247 PBRT patients evaluable for statistical analyses.

All patients received standard comprehensive uveal melanoma evaluation, detailed in the prior literature [21,22,23,24]. All PBRT patients (*n* = 240) included in the analysis were treated with our current standard protocol of 56 gray equivalent (GyE; proton relative biological effectiveness, RBE = 1.1) in 4 fractions over 4 days. Median dose for He^+^ ion RT was 70 GyE in 5 fractions (range, 48–80 GyE, RBE = 1.3). Patients were followed post-treatment in a standardized manner, including complete clinical evaluation, fundus photographs, and ultrasonography post-RT at approximately 3 months, 6 months, and then yearly, on average. We conducted a retrospective review of the patients’ medical records to document treatment outcomes, survival, and complications between August 2020 and August 2021. This study was performed in accordance with the Declaration of Helsinki and with the University of California, San Francisco’s Institutional Review Board’s approval.

### 2.2. Radiation Technique

The tumor base and spatial coordinates were outlined by tantalum markers placed intraoperatively at 4 scleral points. The spatial coordinates were obtained from 2 orthogonal X-ray films. Treatment utilized a customized facemask and dental bite block or chin rest for immobilization. EYEPLAN^®^ software (v3.06, Clatterbridge, UK) was used for treatment planning, including eye and tumor modeling, gaze angle optimization, aperture design, beam parameter delineation, dose–volume histogram (DVH) analysis, marker alignment, and daily treatment positioning.

A 2.5–3 mm lateral aperture perimeter was placed around the tumor projection in the beam’s-eye view, representing the 50% isodose line. This aperture margin accounts for possible microscopic tumor extension, minor patient setup error or movement, and beam penumbra. The distal margin on the tumor range was 3–4 mm, per standard protocol.

To reduce dose delivery to critical structures, modifications, including adjusting the gaze angle to minimize dose to the lens, ciliary body (CB), optic disc, and/or macula, were made when safe and clinically appropriate. The aperture margin at the lens, macula, or disc was focally reduced to 2 mm to reduce the critical-structure dose in certain cases. Similarly, the distal margin was reduced to 2–2.5 mm in particular cases to reduce the dose to the disc, macula, or nerve.

The eye position was monitored throughout the treatment to ensure proper alignment via a closed-circuit television camera system. Custom eyelid retractors helped withdraw the eyelids from the beam path as much as possible. If a significant amount of eyelid remained in the field, then the distal range was adjusted accordingly to compensate for the additional tissue exposure. All patients were treated with a single anterior port using a 67.5-MeV horizontal proton beam generated by a 76-inch isochronous cyclotron or a helium 184” beam.

### 2.3. Statistical Analysis

For each cohort, descriptive statistics were tabulated to characterize patient, tumor, and outcome features. Patient data included age, sex, eye involved, date of treatment, and co-existing medical issues. Tumor size and location were recorded along with the treatment details, such as radiation dose, fractionation, and duration of follow-up.

Clinical outcomes included local control (LC), eye preservation, distant metastasis (DM), and overall survival (OS) recorded post-RT. Durations were calculated from the date of the start of RT until the date of recurrence for local and distant disease, the date of enucleation, and the date of death due to any cause for OS. If the endpoint did not occur, the duration was censored on the date of the last available contact. The probability of LC, ocular retention, being free of DM, and OS were estimated and graphically displayed using the Kaplan–Meier product limit method [25]. Local control is defined as remaining free of local failure post-RT and was recorded when tumor growth (base or thickness) was detected post-RT by clinical examination, A-scan or B-scan ultrasonography, ultrasound biomicroscopy, or serial comparison of fundus photographs and fluorescein angiograms. For patients who had the UM eye enucleated, we recorded the reason for enucleation. Metastatic disease was recorded through clinical and histopathologic examination at the metastatic site. All reported DMs were confirmed to be of UM origin. Overall survival was defined as not dying due to any cause.

A chi-square test was used to evaluate the association between categorical variables, analysis of variance (ANOVA) methods were used to compare subsets for continuous variables, and Pearson’s correlation was calculated to evaluate the relationship between continuous variables. Univariate analyses for time-to-failure outcomes were performed using the log-rank test for categorical variables and Cox’s proportional hazard model using the likelihood ratio (LLR) test for continuous variables. Variables evaluated included gender, age, right/left eye, tumor location, height, largest tumor diameter (LTD), tumor T category, distances to disc and fovea, CB involvement, and dose–volume relationships for the lens, CB, macula, disc, and optic nerve. Multivariate analyses using Cox’s proportional hazard model identified independent predictors of each clinical outcome using a forward stepwise approach with a probability of 0.05 to enter the model and 0.10 to be removed. Only variables not significantly related to each other were considered as predictors in a single analysis, with the model LLR test determining the best fit. Results were summarized by the hazard ratio (HR) and 95% confidence interval (CI) for each predictor in the model. For all analyses, a probability of <0.05 was considered to be statistically significant. Analyses were performed using Stata (v12.1 StatCorp, College Station, TX, USA) and Statistica (v12, StatSoft, Inc., Tulsa, OK, USA) Graphs were generated using Statistica statistical software.

## 3. Results

### 3.1. AYA UM Proton Patient and Tumor Characteristics

The final PBRT cohort included 240 patients treated with 56 GyE in four fractions. Of 247 PBRT patients meeting inclusion criteria, 7 early patients treated between 1994 and 1995 were excluded from the analysis due to the lower RT dose (48 GyE in four fractions). The patient and tumor features of the UM PBRT cohort are shown in Table 1. The median follow-up was 85.7 months (range, 3–301 months). Of the 240 patients, 47.5% were male, and 55% of tumors affected the left eye. The median age at RT was 38.3 years (13.3–45.9), the median tumor height was 4.5 mm (0.8–15.8), and the median LTD was 10.5 mm (2.3–25.1).

Of the 240 patients, 119 (49.6%) had an FNA biopsy at the time of initial treatment. Of the accessible pathology results (*n* = 98), 68 (69.4% of 98) were confirmed as spindle cell/low-grade, 21 (21.9%) had epithelioid features, and 9 (9.2%) had mixed-cell features. Of the specific gene expression profiling (GEP) results available (*n* = 64), 38 (59.4% of 64) patients had class 1A tumors, 12 (18.8%) had type 1B, and 14 (21.9%) had type 2 tumors. Next-generation sequencing for 13 patients showed that the specific somatic genetic mutations were most commonly GNAQ (*n* = 6), SF3B1 (*n* = 5), EIF1AX (*n* = 3), and GNA11 (*n* = 3). Three pathogenic germline mutations were identified in two patients and included CDH1 (*n* = 1), PABL2 (*n* = 1), and NF1 (*n* = 1).

The primary tumor location was 87% choroidal, 12% ciliary body, and 1% iris only. Patients with ciliary body tumors were more likely to have higher category tumors (*p* < 0.001); choroidal tumors had a significantly shorter tumor height and LTD than ciliary body tumors (*p* = 0.02 for each). Of patients with ciliary body tumors (*n* = 20), 65% were over 40 years of age at RT (*p* = 0.015).

The LTD was significantly correlated with the tumor height (r = 0.66, *p* < 0.001). The median tumor distances to the fovea and disc were 2.0 mm and 3.2 mm, respectively, with 52% of tumors within 0–2 mm of the fovea and 41% within 0–2 mm of the disc. There was no relationship between age and any of the distances or dimensions. Table 2 shows the comparison of tumor dimensions by age.

### 3.2. Local Control PBRT

**Figure 1:**
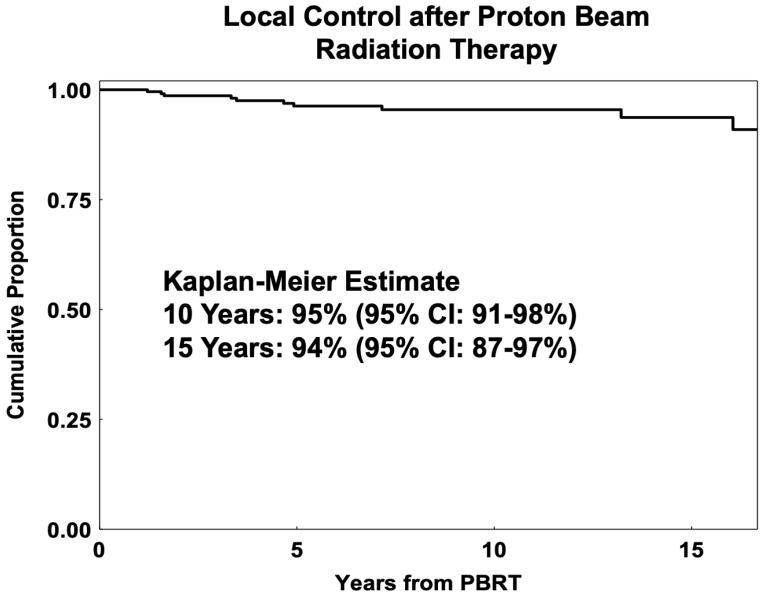
Kaplan–Meier estimates of local control for young-adult and adolescent (age 13 to 45) uveal melanoma patients treated with proton beam radiation therapy (PBRT).

Figure 1 shows post-radiation Kaplan–Meier LC estimates for proton-irradiated AYA patients at RT. In total, 11 patients had a local recurrence (LR) in the PBRT cohort, with a 5-year LC of 96% (92–98%), 10-year LC of 95% (95% CI: 91–98%), and 15-year LC of 94% (95% CI: 87–97%).

With the univariate analysis, patients with ciliary body tumors alone showed a lower LC (*p* = 0.01) than those with choroidal tumors. Patients with tumors closer to the fovea (0–2 mm vs. >2 mm) had better local control (*p* = 0.02). As a continuous variable, increasing LTD was associated with an increased likelihood of local recurrence (*p* = 0.017).

In the multivariate analysis, LTD was the only independent predictor of LC, with greater LTD having lower LC (HR = 1.19, 95% CI: 1.04–1.36, *p* = 0.017). For patients with no iris involvement (*n* = 229), tumors closer to the fovea (≤2 mm vs. ≥2 mm) had better local control (HR = 5.53, 95% CI: 1.19–25.65; *p* = 0.01). The PBRT cohort had limited late failures, with three patients having very late local failures at 13.2, 16.1, and 17.5 years, with all three tumors showing spindle-cell cytology on biopsy (one at initial diagnosis and two at recurrence).

### 3.3. Eye Preservation in PBRT

The 5-year and 10-year eye preservation in the PBRT cohort was 85% (95% CI: 79–89%) and 83% (95% CI: 77–88%), respectively. Table 3 summarizes the variables predicting improved eye preservation. In total, 84% of all enucleations (31 out of 37) occurred within five years post-PBRT. Specifically, the time to enucleation after proton therapy ranged from 0.3 to 17.7 years, with 16 patients undergoing enucleation within two years of RT, 31 patients within five years of RT, and 6 patients after five years of RT. The reasons for enucleation included painful eye in 24 (65%) patients, frequently secondary to neovascular glaucoma, and local recurrence in 10 (27%) eyes. One eye (3%) was enucleated due to presumed LR, but there was no evidence of LR on pathologic examination. The reason for enucleation was unknown in 2 eyes (5%), but there was no evidence of LR in either eye.

**Figure 2:**
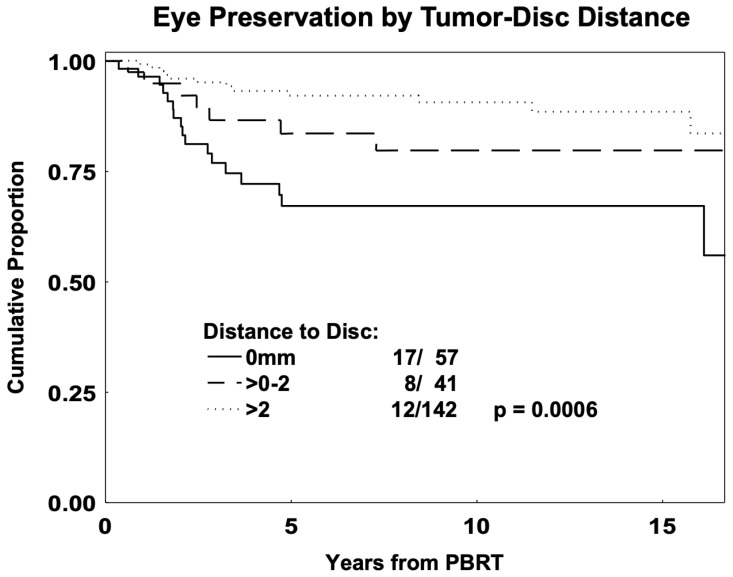
Kaplan–Meier estimates of eye preservation by tumor–disc distance for young-adult and adolescent (age 13 to 45) patients with uveal melanoma (*n* = 240) treated with proton-beam radiation therapy (PBRT).

Based on the American Joint Committee on Cancer classification [26], the UM T-categories were 95 T1 (39.6%), 89 T2 (37.1%), 46 T3 (19.2%), and 10 T4 (4.2%). Patients with category T1–T2 tumors showed better eye preservation than those with category T3–T4 (*p* = 0.002). Patients with increased tumor-to-disc distance (0–2 vs. >2 mm) had better eye preservation (*p* = 0.0007). Univariately, decreased enucleation was predicted by decreased tumor height (HR = 1.21, 95% CI: 1.10–1.33; *p* = 0.0003) and increased tumor–disc distance (HR = 0.32, 95% CI: 0.16–0.65; *p* = 0.0008), as shown in Table 3. Improved eye preservation was also observed with certain tissue dose parameters (Table 3), including ≤50% dose to the lens (*p* = 0.004), disc (*p* = 0.001), nerve length (*p* = 0.0004), macula (*p* = 0.06), and <20% CB volume (*p* = 0.0002) (LLR tests). Figure 2 shows eye preservation by distance to disc.

Using multivariate methods (Table 3), decreased tumor height (HR = 1.12, 95% CI: 1.01–1.2, *p* = 0.036), decreased dose to 20% to the ciliary body (HR = 1.04, 95% CI: 1.01–1.06, *p* = 0.0002), and tumor distance to disc > 2 mm (HR = 0.27, 95% CI: 0.13–0.54, *p* = 0.0003) were independent predictors of eye preservation. For patients without iris involvement, decreased dose to 20% to the ciliary body (*p* = 0.0001) and distance to the disc > 2 mm (*p* = 0.0008) were independent predictors of eye preservation.

### 3.4. Distant Metastasis and Overall Survival

The 5-, 10-, and 15-year DM-free rates in the PBRT cohort were 87% (95% CI: 82–91%), 81% (95% CI: 74–86%), and 73% (95% CI: 64–80%), respectively. The 5-, 10-, and 15-year OS rates were 90% (95% CI: 85–94%), 84% (95% CI: 78–89%), and 76% (95% CI: 67–83%). Of the 240 patients at the time of the last available follow-up during the study, 189 were alive without recurrence, 6 were alive after LR, 6 were alive after DM, 2 were alive after LR and DM, 3 died without any recurrence, 3 died after LR and DM, and 31 died after DM.

**Figure 3:**
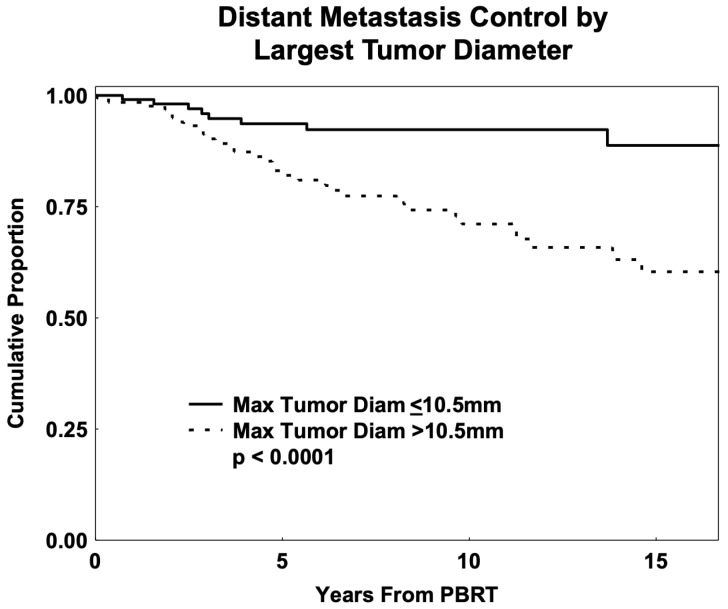
Kaplan–Meier estimates of distant metastasis control by largest tumor diameter for young-adult and adolescent (age 13 to 45) patients with uveal melanoma (*n* = 240) treated with proton-beam radiation therapy (PBRT).

Univariate analysis indicated that patients with T1 tumors had better overall survival than those with category T2–T4 UMs (*p* = 0.0006). Patients with early-category tumors also had decreased DM (*p* < 0.001). Patients with choroidal tumors had better overall survival (*p* = 0.037) and decreased risk of DM (*p* = 0.02) than those with ciliary body tumors without iris involvement. None of the 11 patients with tumors invading the iris (8 with CB involvement and 3 with iris alone) died or had DM recurrence. The median OS for these patients was 83 months (range, 5 to 215). Patients with tumors closer to the fovea (0–2 mm vs. >2 mm) had better overall survival (*p* = 0.007) and decreased DM (*p* = 0.01). Increased LTD was associated with decreased overall survival (HR = 1.15, 95% CI: 1.07–1.24; *p* = 0.0006) and decreased DM control (HR = 1.17, 95% CI: 1.09–1.25; *p* < 0.0001). Figure 3 shows the proportion of patients free of DM by LTD.

The most significant predictor of overall survival was T category, with patients with T1-category tumors having better overall survival compared to those with T2, T3, and T4 tumors combined (HR = 4.04, 95% CI: 1.69–9.70; *p* = 0.0003). T category was also the most significant predictor of DM (*p* = 0.0001), with T1-category tumors having better DM control compared with category T2–T4 (HR = 3.65, 95% CI: 1.28–10.40; *p* < 0.0001) followed by better DM control with a decreased LTD (HR = 1.09, 95% CI: 1.01–1.20, *p* = 0.04). The age ≤ 30 subgroup (*n* = 46) had a 5-year DM-free rate of 97% and had only 4% CB tumors.

### 3.5. Helium-Ion RT Cohort

Patient and tumor features for the Helium cohort are summarized in Table 4. In total, 78 patients were treated between 1979 and 1992. There were slightly more female (53%) than male patients (47%), and tumors were found mostly in the left eye (56% left vs. 44% right). The Helium cohort’s median follow-up was 256.7 months (24–450). The median age at RT was 37.1 (18.1–45.5), the tumor height was 6.0 mm (2.9–14), the LTD was 10.5 mm (5–25), the distance to disc/fovea was 3.5/3.0 mm, and 22% had tumors with ciliary body involvement. The LTD was larger for ciliary body tumors (*p* < 0.0001) and smaller for tumors near the disc (*p* = 0.03) or fovea (*p* < 0.0001). The median (range) dose for helium RT was 70 GyE (48–80).

**Figure 4:**
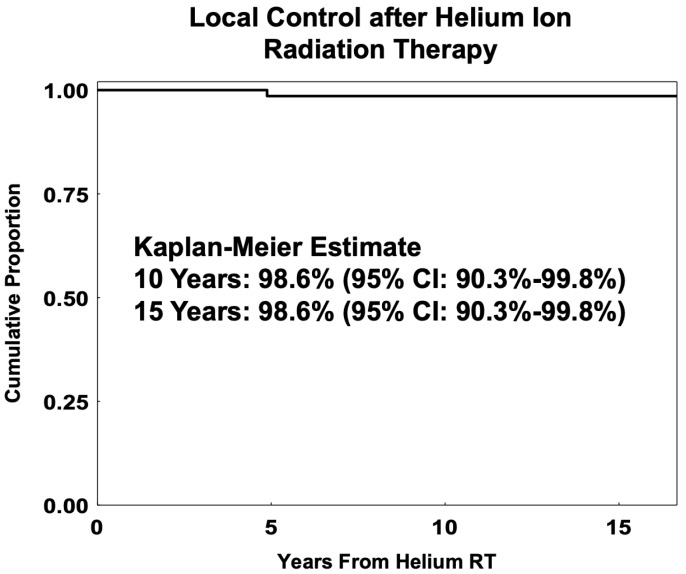
Kaplan–Meier estimates of local control for young-adult and adolescent (age 13 to 45) patients with uveal melanoma (*n* = 78) treated with helium ion radiation therapy (RT).

The Helium cohort had a 5-, 10-, and 15-year LC of 98.6% (95% CI: 90.3–99.8%) with only one failure at 58.6 months after helium-ion RT. Figure 4 shows the Kaplan–Meier curves for local control after helium-ion RT. The 5- and 10-year eye preservation rates were 84% (95% CI: 74–91%) and 82% (95% CI: 71–89%), respectively. The 5-, 10-, and 15-year estimates of overall survival were 90% (95% CI: 81–95%), 86% (95% CI: 76–92%), and 83% (95% CI: 73–90%), respectively. There were a total of 21 deaths in the Helium cohort; 17 patients died with distant metastases, and 4 died without recurrence or DM. The 5-, 10-, and 15-year estimates of DM-free survival were 87% (95% CI: 77–93%), 85% (95% CI: 74–91%), and 79% (95% CI: 68–87%), respectively.

## 4. Discussion

Uveal melanoma presents a rare and challenging diagnosis for adolescents and young adults, considering the potential for dramatic implications on vision, quality of life, and life expectancy [1,9,27,28]. Uveal melanoma typically presents in advanced age, and the unique characteristics and outcomes of younger patients may aid in personalized care for this subset of patients. Limited long-term data exist on post-particle-beam radiation for this age group, though there is increasing awareness of the need to study the cancer course and outcomes in AYAs [3,4,7,11,13]. Additionally, outcomes for AYAs are frequently grouped with a broader adult category, masking unique characteristics of this understudied population [29]. In this work, the study presented at the American Society for Radiation Oncology meeting [30] is expanded upon. Our study adds to this body of work by including a detailed analysis of long-term outcomes for AYAs with UM treated with proton and helium-ion RT. This age group had a more favorable genetic and cytologic UM profile and demonstrated excellent 15-year local control (94% PBRT, 99% helium RT) and higher 15-year overall survival (76% PBRT, 83% helium RT) post-particle RT compared to older UM patients.

Local control was high for proton-irradiated AYA patients, with LC at 95% at 10 years and 94% at 15 years. These results are consistent with the limited current research in young UM populations [3,4,5,11]. In our population, greater LTD was the most important predictor for lower LC. Ciliary body tumors tended to have a higher T category, larger size, and lower local control in our young patient cohort. The incidence of CB tumors increased with age and was more commonly seen in patients > 40 years of age at RT. For non-iris tumors, those closer to the fovea were smaller and had better LC, OS, and DM control, likely due to earlier detection with visual disturbance. Additionally, considering that T1-category tumors have significantly better OS and DM control, it is imperative to develop risk-assessment tools to identify AYAs at risk of UM and introduce regular screening and earlier intervention to reduce the disease burden in this age group [7,31]. A recent case-control study identified novel risk factors and confirmed established risk factors for AYAs with UM; thus, continued research is critical to delineate particular characteristics of this understudied population [7].

The PBRT dose was consistent at 56 GyE in four fractions, resulting in high local control; however, seven early proton patients not included in the analysis received 48 GyE total in four fractions and had local recurrences. The institutional team had made the empirical clinical adjustment to 56 GyE due to these early failures. At Massachusetts General Hospital (Boston, MA, USA), the standard dose is 70 GyE in five consecutive daily fractions, and small posterior macular tumors may be given 50 GyE in five fractions [32]. A nonrandomized study at Lawrence–Berkeley Laboratory (median follow-up 8.5 years) found that reduction from 80 GyE in five fractions to 48 GyE in four fractions with helium resulted in no LC difference (96%) at 80, 70, 60, or 50 GyE in five fractions [33]. However, LC fell to 87% with 48 GyE in four fractions [33]. If 50 GyE in five fractions is utilized for non-macular tumors at select institutions, the biologic equivalence of 48 GyE/four fractions and 50 GyE/five fractions may need to be further studied, and local control should be followed long-term for young UM patients [34].

Five- and 10-year eye preservation rates in our PBRT cohort were 85% and 83%, respectively, with most enucleations (84%) occurring in the first 5 years post-PBRT. The average overall enucleation rate for general UM patients is ~10% (range, 0–25%) at 5 years and ~15% at 15 years [35,36]. The most significant factors predicting eye preservation were decreased dose to 20% of the ciliary body, distance to disc > 2 mm, and decreased tumor height. Similarly, dose–volume parameters at the 50% dose level to the nerve/disc have shown an important association with side effects, such as neovascular glaucoma and enucleation [20,21]. Utilizing such parameters can assist in communicating patient prognosis and in treatment planning choices regarding margins and normal tissue-sparing techniques.

The 10-year DM free rate in our PBRT cohort (81%) is consistent with Pica et al.’s proton data showing a similar 10-year DM free rate of 80.3% in patients aged 15–39 [11]. In our cohort, the 10-year OS rate was 84%, reflecting the lower metastatic rate compared to older adults and close follow-up after metastatic diagnosis. Although our group had a lower 10-year OS rate (84%) than reported for the AYAs by Pica et al. (94.6%) [11], the Pica et al. comparative data may have under-reporting of deaths, given that the 10-year metastatic incidence was 19.7%, potentially due to their international population and geographical circumstances [37]. The OS rate was well within the range of other younger UM patient studies post-plaque and proton irradiation (10-year rate of 52–100%), most of which have much smaller cohort sizes [3,4,5,6,9,10,11,12,13,15,38].

Analysis showed LTD as a significant predictor for DM, similar to Kaliki et al. [19]. Using multivariate methods, tumor T category was the most significant predictor of DM in our cohort. This was followed by LTD. The age ≤ 30 subgroup had a 5-year DM-free rate of 97% and had only 4% CB tumors. Previous UM reports on children and teenagers had found better metastatic prognosis in patients under 16–20 years of age [3,4,10,13,19]. A study of 8033 patients showed smaller tumor dimensions and lower tumor-related metastasis rate at 10 years: 9% (age ≤ 20) vs. 23% (age 21–60) vs. 28% (age > 60) [10]. The recent age-matched study (15–39 vs. >40 years) reported that LC, DM, and OS rates were similar [11], in contrast to these prior studies showing improved survival in younger UM series.

The modern workup of uveal melanoma includes FNA biopsy for risk stratification [39,40,41]. Among the PBRT cohort, approximately half had an FNA biopsy. The majority of UMs in this cohort had spindle-cell cytology, contrary to the cytologic distribution reported for the general UM population, with most tumors generally being of mixed or epithelioid features [42,43]. Epithelioid cytology is an established predictor of poor prognosis [42,43,44] but was identified in only 21.4% of the tested cohort. Additionally, the majority of the tested patients in our cohort had GEP class 1 tumors, which have a low metastatic risk [44]. These findings suggest that UM in AYAs may have a more favorable cytologic portfolio and, by extension, better prognosis, although for unclear reasons. These results require validation in larger cohorts, and future research may further delineate genetic patterns, risk factors, and potential therapeutic targets to support younger UM patients better.

The Helium cohort had a similar age distribution and tumor dimensions to those of the PBRT group. The group exhibited excellent 15-year LC at 99%, 10-year eye preservation of 82%, and 15-year OS rate of 83%, similar to the PBRT patients. No other similar study exists for younger helium-RT-treated UM patients.

The limitations of this study are the retrospective nature of the report and the lack of an age-adjusted cohort for comparison. The survival estimates may be overestimated since we relied on the availability of information in the medical records for DM and death reports. However, given the rarity of this disease and the importance of further study in this unique population, the current study provides insight into an important subset of patients. Further work in genetic testing and molecular therapeutic targets needs to be assessed for UM patients, specifically younger patients at risk for metastatic disease.

## 5. Conclusions

Excellent long-term local control (at 15 years, 94% for PBRT and 99% for Helium cohort) and good eye preservation (at 10 years, 83% for PBRT and 82% for Helium cohort) are seen in AYA UM patients aged 13 to 45 treated with proton and helium-ion radiation. Long-term survival rates were comparable to those reported in multiple other younger cohorts, and adults under 30 demonstrated notably higher DM-free survival. Tumor height, tumor–disc distance, and decreased dose to the ciliary body were independent predictors of eye preservation. Thus, specific clinical and dose–volume parameters may be used to assess patient prognosis, aid in treatment planning, and anticipate secondary complications. LTD and tumor T category were the most important predictors of OS and DM control, underscoring the need for early detection through developing and implementing standardized risk-assessment tools for UM, particularly in AYAs. To our knowledge, this report is one of the few long-term studies focusing on the adolescent and young-adult UM age group post-proton treatment and is the first study of this age group in helium-ion treatment. Results demonstrate particle therapy to be an effective therapy for younger UM patients, and further study into the genetic markers, risk factors, quality of life, and long-term effects of treatment may add to the personalized care of younger patients with uveal melanoma.

## Figures and Tables

**Table 1:** Features of uveal melanoma (UM) and characteristics of adolescents and young adults (age 13 to 45) with UM treated with proton-beam radiation therapy (*n* = 240).

	Median	Range
Follow-Up (months)	85.7	3–301
Age at RT (years)	38.3	13.3–45.9
Tumor Height (mm)	4.5	0.8–15.8
Largest Tumor Diameter (mm)	10.5	2.3–25.1
Tumor Distance to Disc (mm)	3.2	0–24.0
Tumor Distance to Fovea (mm)	2.0	0–24.0
%Tumor 0–2 mm of Disc	41%	
%Tumor 0–2 mm of Fovea	52%	
%Ciliary Body Tumors	11%	

Abbreviations: GyE = gray equivalent; RT = radiation therapy.

**Table 2:** Comparison of tumor dimensions in the proton-beam radiation therapy (PBRT) and helium-ion RT cohorts by age: mean (SD).

PBRT	≤30 *n* = 46	>30–35 *n* = 43	>35–40 *n* = 59	>40–45 *n* = 92	ANOVA Probability Value
Height	5.5 (3.2)	5.3 (2.5)	4.6 (2.4)	5.5 (3.1)	*p* = 0.24
Max Diameter	11.4 (4.6)	10.3 (2.9)	10.4 (3.7)	11.0 (3.9)	*p* = 0.41
**Helium** **-** **Ion RT**	**≤30** ***n* = 14**	**>30–35** ***n* = 19**	**>35–40** ***n* = 18**	**>40–45** ***n* = 27**	**Kruskal–Wallis** **Probability Value**
Height	5.5 (3.2)	5.3 (2.5)	4.6 (2.4)	5.5 (3.1)	*p* = 0.30
Max Diameter	11.4 (4.6)	10.3 (2.9)	10.4 (3.7)	11.0 (3.9)	*p* = 0.74

Abbreviations: ANOVA = analysis of variance, SD = standard deviation.

**Table 3:** Variables predicting improved eye preservation for adolescent and young-adult (age 13 to 45) patients with uveal melanoma (UM) treated with proton-beam radiation therapy.

Significant Univariate Predictors	Hazard Ratio (95% CI)	LLR Test Probability Value
↓ Tumor Height	1.21 (1.10–1.33)	0.0003
↑ Tumor–Disc Distance (≤2 vs. >2 mm)	0.32 (0.16–0.65)	0.0008
↓ Dose to:		
≤50% Nerve	1.17 (1.07–1.26)	0.0004
≤ 50% Lens	1.02 (1.01–1.03)	0.004
≤50% Disc	1.01 (1.004–1.02)	0.001
≤20% Ciliary Body	1.04 (1.02–1.06)	0.0002
**Multivariate Independent Predictors**		
↓ Tumor Height	1.12 (1.01–1.25)	0.036
↓ ≤20% Dose to Ciliary Body	1.04 (1.01–1.06)	0.0002
↑ Tumor–Disc Distance (≤2 vs. >2 mm)	0.27 (0.13–0.54)	0.0003

Abbreviations: CI = confidence interval; LLR = likelihood ratio, ↑ = increasing, ↓ = decreasing

**Table 4:** Characteristics of adolescents and young adults (age 13 to 45) with uveal melanoma (UM) treated with helium-ion radiation therapy (*n* = 78) and UM features.

	Median	Range
Follow-Up (months)	256.7	24–450
Age at RT (years)	37.1	18.1–45.5
Median Dose (GyE)	70	48–80
Tumor Height (mm)	6.0	2.9–14.0
Largest Tumor Diameter (mm)	10.5	5.0–25.0
Tumor Distance to Disc (mm)	3.5	0–13.0
Tumor Distance to Fovea (mm)	3.0	0–10.5
%Tumor 0–2 mm of Disc	33%	
%Tumor 0–2 mm of Fovea	44%	
%Ciliary Body Tumors	22%	

Abbreviations: GyE = gray equivalent; RT = radiation therapy.

## Data Availability

Data from this study are available via the corresponding author upon reasonable request.

## Abbreviations

The following abbreviations are used in this manuscript:

ANOVA	Analysis of variance
AYA	Adolescent and young-adult
CB	Ciliary body
CI	Confidence interval
DM	Distant metastasis
DVH	Dose–volume histogram
GEP	Gene expression profiling
GyE	Gray equivalent
HR	Hazard ratio
LC	Local control
LLR	Likelihood ratio
LR	Local recurrence
LTD	Largest tumor diameter
OS	Overall survival
PBRT	Proton-beam radiation therapy
RBE	Relative biological effectiveness
RT	Radiation therapy
UM	Uveal melanoma
